# Digital image analysis and machine learning-assisted prediction of neoadjuvant chemotherapy response in triple-negative breast cancer

**DOI:** 10.1186/s13058-023-01752-y

**Published:** 2024-01-18

**Authors:** Timothy B. Fisher, Geetanjali Saini, T. S. Rekha, Jayashree Krishnamurthy, Shristi Bhattarai, Grace Callagy, Mark Webber, Emiel A. M. Janssen, Jun Kong, Ritu Aneja

**Affiliations:** 1 Department of Biology, Georgia State University, Atlanta, GA 30302 USA; 2 School of Health Professions, University of Alabama at Birmingham, Birmingham, AL 35294 USA; 3 JSSAHER (JSS Academy of Higher Education and Research) Medical College, Mysuru, Karnataka India; 4 Discipline of Pathology, University of Galway, Galway, Ireland; 5 Department of Pathology, Stavanger University Hospital, Stavanger, Norway; 6 Department of Mathematics and Statistics, Georgia State University, Atlanta, GA 30303 USA; 7 Department of Chemistry, Bioscience and Environmental Engineering, University of Stavanger, Stavanger, Norway

**Keywords:** Triple-negative breast cancer, Neoadjuvant chemotherapy, Machine learning, Digital image analysis, Feature engineering

## Abstract

**Background:**

Pathological complete response (pCR) is associated with favorable prognosis in patients with triple-negative breast cancer (TNBC). However, only 30–40% of TNBC patients treated with neoadjuvant chemotherapy (NAC) show pCR, while the remaining 60–70% show residual disease (RD). The role of the tumor microenvironment in NAC response in patients with TNBC remains unclear. In this study, we developed a machine learning-based two-step pipeline to distinguish between various histological components in hematoxylin and eosin (H&E)-stained whole slide images (WSIs) of TNBC tissue biopsies and to identify histological features that can predict NAC response.

**Methods:**

H&E-stained WSIs of treatment-naïve biopsies from 85 patients (51 with pCR and 34 with RD) of the model development cohort and 79 patients (41 with pCR and 38 with RD) of the validation cohort were separated through a stratified eightfold cross-validation strategy for the first step and leave-one-out cross-validation strategy for the second step. A tile-level histology label prediction pipeline and four machine-learning classifiers were used to analyze 468,043 tiles of WSIs. The best-trained classifier used 55 texture features from each tile to produce a probability profile during testing. The predicted histology classes were used to generate a histology classification map of the spatial distributions of different tissue regions. A patient-level NAC response prediction pipeline was trained with features derived from paired histology classification maps. The top graph-based features capturing the relevant spatial information across the different histological classes were provided to the radial basis function kernel support vector machine (rbfSVM) classifier for NAC treatment response prediction.

**Results:**

The tile-level prediction pipeline achieved 86.72% accuracy for histology class classification, while the patient-level pipeline achieved 83.53% NAC response (pCR vs. RD) prediction accuracy of the model development cohort. The model was validated with an independent cohort with tile histology validation accuracy of 83.59% and NAC prediction accuracy of 81.01%. The histological class pairs with the strongest NAC response predictive ability were tumor and tumor tumor-infiltrating lymphocytes for pCR and microvessel density and polyploid giant cancer cells for RD.

**Conclusion:**

Our machine learning pipeline can robustly identify clinically relevant histological classes that predict NAC response in TNBC patients and may help guide patient selection for NAC treatment.

**Supplementary Information:**

The online version contains supplementary material available at 10.1186/s13058-023-01752-y.

## Introduction

Triple-negative breast cancer (TNBC) is an aggressive breast cancer subtype that lacks expression of estrogen, progesterone, and human epidermal growth factor 2 receptors [1]. TNBC accounts for 15–20% of all breast cancers, affecting nearly half a million women in the USA each year [2, 3]. The 5-year survival rate of TNBC patients is 15% lower than that of patients with other breast cancer subtypes [1]. At the time of diagnosis, TNBCs tend to have a more advanced histologic grade and larger size compared to hormone-positive breast cancers [4]. TNBCs also have higher recurrence and metastasis rates and typically metastasize to the brain, lung, and liver [5, 6]. No targeted or endocrine therapy is available for TNBC, and neoadjuvant chemotherapy (NAC) is the standard of care. NAC involves the use of chemotherapy prior to surgery to reduce tumor size, downgrade tumors amenable to resection, and improve long-term clinical outcomes. The primary endpoint of NAC is a pathological complete response (pCR), defined as the absence of residual invasive disease (RD) in the breast and axilla.

pCR is an important predictor of disease-free survival and overall survival in patients with TNBC [7]. Only 30–40% of TNBC patients achieve pCR with conventional NAC; the rest (~ 70%) either do not respond or respond partially to NAC. Non-responders and partial responders can be spared treatment side effects and offered alternative treatment regimens (e.g., a combination of NAC and immunotherapy) to improve outcomes and decrease morbidity [8–10]. More recently, immunotherapy has shown success in TNBC management, and the FDA has approved pembrolizumab for use in combination with NAC in high-risk patients with early-stage TNBC [11].

Although the mechanisms underlying chemoresistance in TNBCs remain elusive, the marked inter- and intratumoral heterogeneity in TNBCs may contribute to variability in NAC response. Currently, there is a lack of multi-modal biomarkers that can stratify TNBC patients into NAC responders, partial or non-responders, hindering personalized approaches for TNBC management. Furthermore, there is limited information on the robustness and accuracy of current biomarkers, e.g., Ki67, pH3, tumor-infiltrating lymphocytes (TILs), and histological features in predicting NAC treatment response individually or in combination. Traditional staining techniques provide limited information about the immune landscape (e.g., type of TILs). The low reproducibility and objectivity of traditional scoring methods also impair the clinical adoption of these markers. TNBCs are heterogeneous, and their tumor microenvironment (TME) represents a complex ecosystem of cellular components, such as tumor, stromal, and immune cells. Communication between TME components and their spatial relationships affect cancer progression, treatment response, and disease outcomes [12, 13]. Studies have shown that the histomorphological components of the TME, such as a tumor, microvessels (MVD), polyploid giant cancer cells (PGCCs), immune cells, and necrotic areas, can help predict NAC response in TNBC [14]. Advances in computing, imaging, and pathology have created new opportunities to explore the relationships between histology, molecular events, and clinical outcomes to help predict NAC response in patients with TNBC [15].

Manual histomorphological characterizations of hematoxylin and eosin (H&E)-stained tissues is time-consuming and prone to inter- and intra-observer variability and fails to capture the TME spatial architecture, limiting their clinical value. Machine learning (ML) can more accurately and efficiently characterize the TME [16–18]. ML outperforms humans in terms of accuracy and speed and can identify novel predictive features and spatial patterns beyond human recognition [16–18]. The aim of this study was to develop an ML-based model to effectively predict NAC response (pCR or RD) in TNBC patients using spatial histological features from whole slide images (WSIs) of H&E-stained biopsy tissue sections.

## Methods

### Study population

H&E-stained pre-NAC core needle biopsies from treatment-naïve patients with TNBC were acquired from the Decatur Hospital, Georgia, USA, and the University of Galway, Ireland. The Decatur cohort was used as a discovery cohort for model development, and the Galway cohort was used as a validation cohort for the developed model. Patient samples with little to no tissue area, staining issues, or plating artifacts were excluded from the analysis. After this screening process, the final sample sizes of the model development and validation cohorts for prediction analyses were 85 and 79, respectively [19]. Patient clinical information of the Emory and Galway cohorts is presented in Table 1.

**Table 1:** Clinical information of the Emory Hospital and Galway cohorts

	Emory hospital dataset (*n* = 85)	Galway dataset (*n* = 79)
*Age*		
< 55 years old	37	47
= > 55 years old	48	31
NA	0	1
*Grade*		
1	3	0
2	18	21
3	66	58
*Tubule formation (T)*		
1	1	0
2	3	5
3	52	74
NA	29	0
*Nuclear pleomorphism (P)*		
1	2	0
2	7	4
3	47	75
NA	29	0
*Mitotic count (M)*		
1	6	21
2	16	28
3	34	30
NA	29	0
*Vital status*		
Alive	53	69
Dead	32	9
NA	0	1

*NA* Non-available

### Tumor slide selection and annotation

**Fig. 1:**
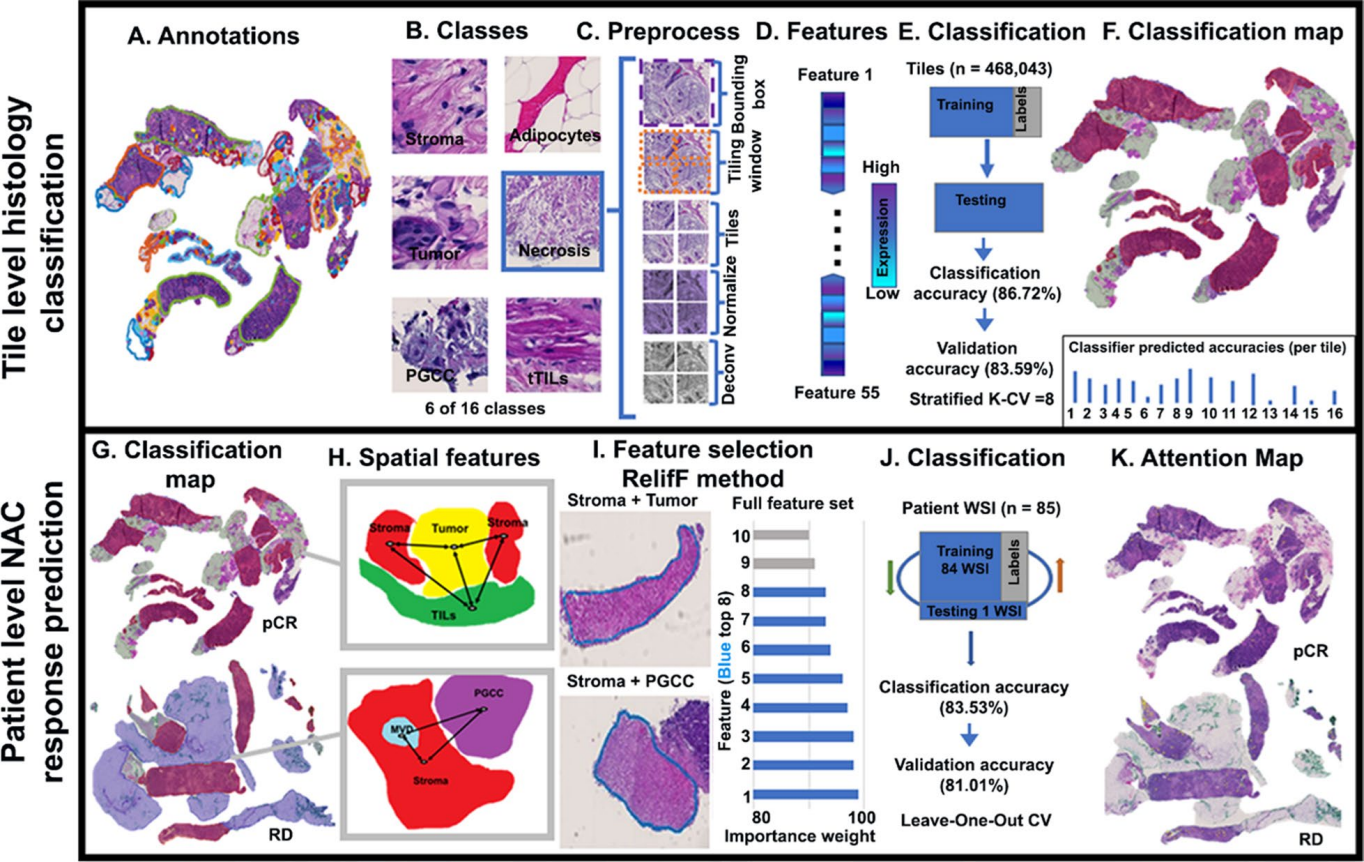
Overall schema of the developed NAC response prediction pipeline. The tile-level histology classification module (first step) consists of **A** training WSI annotation; **B** definition of histology classes of interest; **C** tile preprocessing; **D** feature extraction and selection; **E** classifier training, testing, and validation; and **F** generation of histology classification map. The patient-level NAC response prediction module (second step) consists of **G** graph node identification; **H** TME spatial descriptor computation; **I** graph construction and graph feature selection; **J** machine learning model training, testing, and validation; and **K** generation of an attention map with highlighted tissue regions with full feature set. Abbreviations: FE, feature extraction; sTILs, stromal TILs; tTILs, tumor TILs; Feat, feature; MVD, microvessel; PGCC, polyploid giant cancer cell

**Fig. 2:**
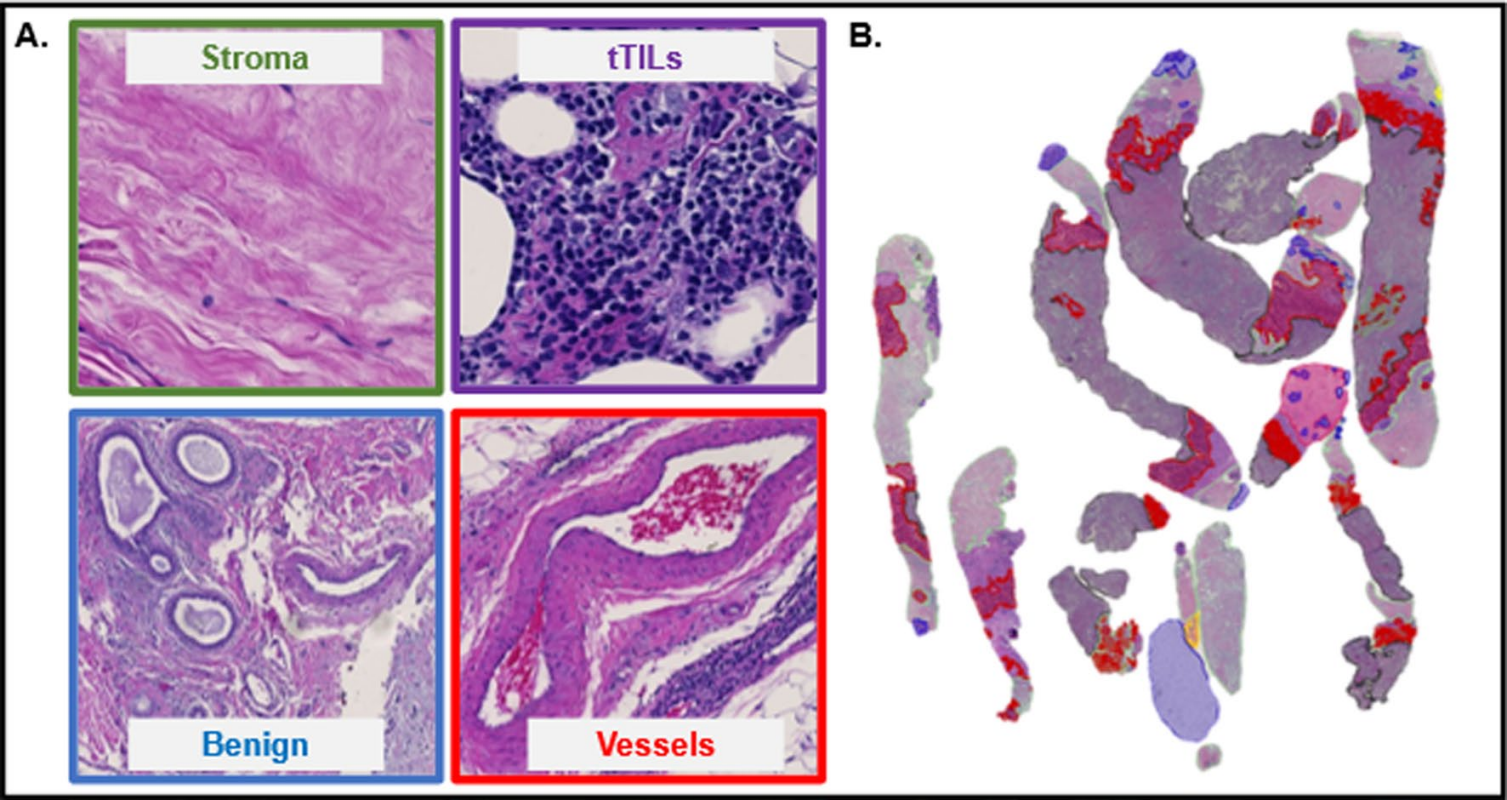
Representative image tiles of distinct histological classes and a tile-level histology classification map. **A** Typical examples of image tiles (224 × 224) capturing stroma, tTILs, benign tissue, and vessels are presented. **B** A histology classification map is presented to visualize the spatial organization of TME components related to stroma (green), benign tumor (blue), tTILs (purple), and blood vessels (red)

H&E-stained slides were scanned by a slide scanner (Hamamatsu NanoZoomer 2.0-HT C9600-13) at 40 × magnification (0.23 μm/pixel). All WSIs were reviewed and annotated by board-certified pathologists at maximum resolution with an open-source image processing software (QuPath, ver. 0.1.2) (Fig. 1A). A total of 16 histology labels were annotated, including tumor, stroma, adipocytes, PGCC, normal tissue, stromaTIL (sTIL), blood vessels, benign tumor, MVD, tertiaryTIL (teTIL), tumorTIL (tTIL), in situ carcinoma, hemorrhage, necrosis, apocrine change, and mucinous change. teTILs are TILs close to tertiary lymphoid structures [20]. Each WSI background was labeled separately. Coordinates and histology class labels of tissue region contours were saved and preprocessed (Figs. 1A, 2B).

### Tile-level preprocessing

Before model training, representative histology regions in WSIs were annotated with contours for each histology class. For each contour, a bounding box was created within the ground-truth area to extract the annotated tissue region. A sliding window of size 224 × 224 pixels was used to partition each WSI into image tiles. Only tiles overlapping the annotated areas by at least 90% were retained (Fig. 1C). The spatial containment query was invoked to identify the histology class for each tile. All image tiles were normalized by the stain color prior to model development [21]. Additionally, the image channel associated with the hematoxylin stain was separated from each color image tile by color deconvolution (Fig. 1C) [22].

### Tile-level histology feature extraction and classification

Tile-level histology features were derived from tissue tiles of different histology classes. After the preprocessing step, 468,043 tiles were produced from WSIs of model development cohort. The tile histology classification performance was evaluated by a stratified eightfold cross-validation strategy [23–25]. Additionally, an independent validation set was established with all tiles from the validation cohort. We extracted 80 tile-level features from each tile by six image texture extraction methods: gray-level co-occurrence matrix (GLCM; Method S1A), Gabor filter (Method S1B), local binary patterns (LBP; Method S1C), Tamura (Method S1D), lower-order histogram (Method S1E), and higher-order histogram (Method S1E) [26–34]. These 80 features were further reduced by excluding features that had 0, not applicable (NA), or repetitive values. We reduced the total number of texture features to 55 by exclusion criteria (Additional file 1: Table S1). Four ML classifiers were used to classify image tiles by the resulting feature set, including 1-nearest neighbor (1NN), linear support vector machine (linSVM), radial basis function SVM (rbfSVM), and ensemble tree (ensembleTree) with the RUSBoost method [35–37]. For model development and validation, the eightfold cross-validation mechanism was used. Each time, seven folds of data were used for training while the remaining one fold was used as the testing set (Fig. 1E). The stratified eightfold cross-validation method ensured that each data fold contained representative samples from each class and reliably assessed the tile histology class prediction performance. For each testing image tile, a trained classifier produced 16 probability values, one for each histology class of interest. Using the predicted tile histology class labels and tile spatial coordinates, we assembled tile-level histology class labels spatially and produced tile-level histology classification maps for each patient (Fig. 2). Each histology class was represented by a unique color in the classification maps, enabling the visualization of TME histology component distributions within a tissue context (Fig. 1F).

### Spatial TME feature extraction and NAC response prediction

The leave-one-out cross-validation (LOO-CV) method was used to evaluate the patient-level NAC response prediction performance [38, 39]. Similar to the tile-level histology class classification step, the model development and validation cohorts were used for model development and validation, respectively. As tumor cells interact closely with immune cells, stroma, PGCCs, and adipocytes in the TME [40–45], histology classification maps were generated for each patient. To better model the spatial relationships of these histology components in biological systems [46–53], we created TME graphs to characterize tissue TME states and the spatial interactions of tissue regions of paired histology classes. For each pair of histology classes (e.g., tumor and PGCC maps), a TME graph was constructed from the corresponding tile-level histology maps. A simple graph $G=(V, E)$ is undirected and unweighted, with V as the graph node set and E as the graph edge set [46, 49]. Each tile cluster was determined as a spatially connected tile component, with all connected tiles sharing the same histology class label. The centroids of the resulting tile clusters were used as nodes in a graph [47]. A graph edge between a pair of nodes $u$ and $v$, i.e., $edge(u, v)$ was established using the Euclidean distance for a given pair of node histology classes. In this way, the spatial histology class distribution was represented by a TME graph structure [46]. Next, a set of TME features was extracted from each TME graph. In total, 20 graph features related to texture feature averages, local node configuration, and global graph connectivity were produced for each patient (Additional file 1: Table S2) [47]. Specifically, the texture feature averages were derived from the tile-level feature set (Method S2A). Local node configuration features were used to characterize the local neighborhood information (Method S2B), while graph connectivity features represented the global graph structures (Method S2C).

For an optimal prediction performance, we retained the top eight TME graph features using the importance weight method. The importance weight of graph features was determined using the ReliefF algorithm, a filter-method approach designed to solve classification problems with discrete or numerical features [54–57]. A feature has a lower importance weight if a feature value difference is observed in a neighboring instance pair with the same histology class (i.e., a ‘hit’). By contrast, a feature presents a higher importance weight if a feature value difference is observed in a neighboring instance pair with different histology classes (i.e., a ‘miss’). The top eight TME graph features ranked by importance weights were retained for patient-level NAC response prediction [58–66].

Four ML classifiers, including 1NN, linSVM, rbfSVM, and ensemble tree with the RUSBoost method, were used for patient-level NAC response prediction. Each patient was represented by the top eight TME graph-derived features associated with histology class pairs. Each classifier produced two NAC response class probability values, one for pCR and the other for RD. A patient was predicted to belong to the NAC response class associated with a larger class probability.

### Statistical analysis

Statistical analysis was performed by Python (Python Software Foundation, https://www.python.org/), MATLAB 2020a (Natick, MA, USA), and R (R Foundation for Statistical Computing, Vienna, Austria, http://www.R-project.org/). The ReliefF importance weights were used to assess the significance of the selected TME graph features [54–56, 67, 68]. The resulting prediction performance was represented by a confusion matrix. The NAC response class pCR and RD were considered positive and negative groups, respectively. A false-positive (FP) was a RD case incorrectly predicted as pCR, while a false-negative (FN) case was a pCR case incorrectly predicted as RD. Multiple evaluation metrics were computed, including accuracy, sensitivity (i.e. Recall), specificity, precision, and F1-Score. The tile-level histology classifier was evaluated by the stratified k-fold cross-validation (*k* = 8), while the patient-level NAC response prediction was assessed by the LOO-CV.

## Results

### ML classifier provides accurate tile-level histology classification in H&E-stained WSIs

**Fig. 3:**
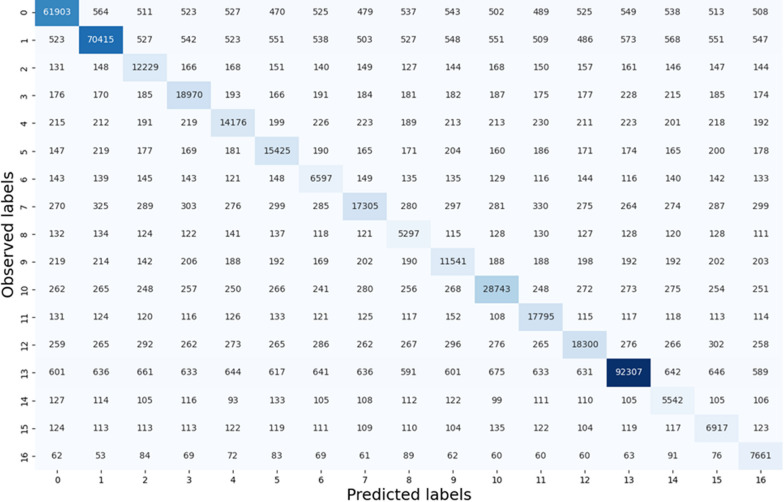
Testing tile-level histology classification performance in the model development cohort. Confusion matrix showing the aggregated performance of the rbfSVM model for tile-level histology class prediction (i.e., 0, stroma; 1, tumor; 2, tertiary TILs; 3, stroma TILs; 4, normal tissue; 5, PGCCs; 6, blood vessels; 7, necrosis; 8, microvessel; 9, benign tumor; 10, tumor TILs; 11, in situ carcinoma; 12, hemorrhage; 13, adipocytes; 14, apocrine change; 15 mucinous change; and 16, background). Abbreviations: 1NN, 1-nearest neighbor; linSVM, linear support vector machine SVM; PGCC: polyploid giant cancer cells; rbfSVM, radial basis function SVM

**Fig. 4:**
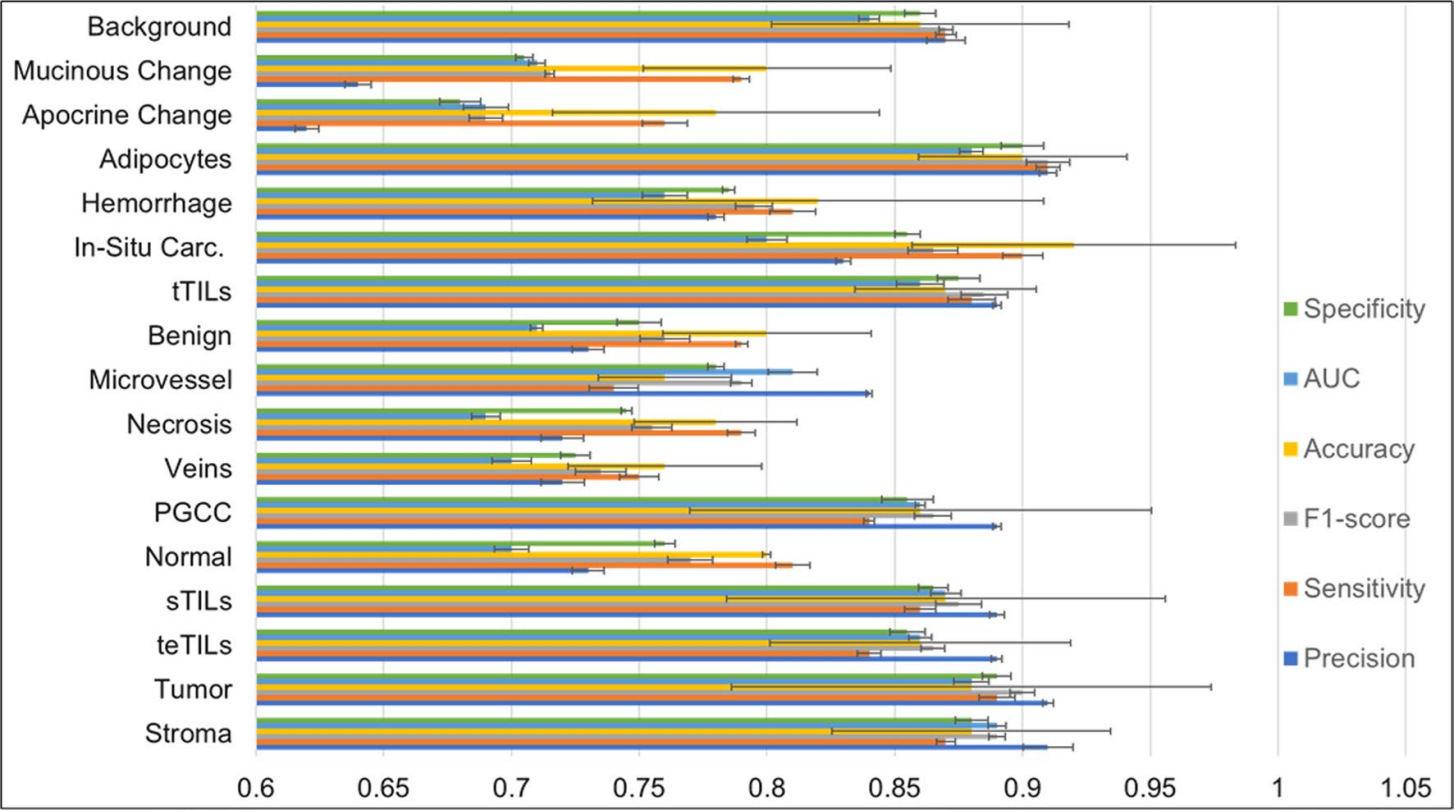
Testing tile-level histology classification performance of the rbfSVM classifier with the Emory Hospital development cohort. Each bar represents the weighted average of tiles and their predicted probabilities during testing for a histology class. Specificity (green) measures how often the rbfSVM classifier correctly predicted true negatives. AUC (blue) reflects the model's ability to distinguish between positive and negative classes. Accuracy (yellow) indicates the proportion of correct predictions out of total predictions. F1-score (gray) presents a balanced view of rbfSVM classifier performance. Sensitivity (orange) suggests how often the rbfSVM classifier correctly identifies positive instances. Precision (blue) indicates how often the rbfSVM classifier correctly predicts true positives. Error bars represent the 95% confidence interval in each case

Our results suggest that the 16 histology classes of interest were well differentiated by 55 tile texture features for the model development cohort (Additional file 1: Table S1). With the stratified eightfold cross-validation strategy, we used onefold of image tiles to train the classifier and test it with the remaining seven folds in each round. The average training accuracies of tile-level histology classification by 1NN, linSVM, and ensemble tree were 63.86%, 61.33%, and 80.08%, respectively. Of the four classifiers that we trained and tested, the best performance was achieved by the rbfSVM with an average training and testing accuracy of 87.16% and 86.72%, respectively (Fig. 3). The individual histology class average testing accuracy ranged from 72.51 to 91.18%. Additionally, the classifier reached an average recall from 75.11 to 92.97%, an average precision from 62.47 to 91.28%, and an average F1-score from 70.21 to 92.81% for all histology classes on the testing dataset (Fig. 4). It was noticed that the rbfSVM classifier was good at recognizing classes such as stroma, tumor and adipocytes, but weak at recognizing apocrine or mucinous change. Detailed classification results with Emory cohort are provided in Additional file 1: Figures S5–S7.

When the best tile-level histology classifier (rbf-SVM), trained using the model development cohort, was applied to the validation cohort, an average validation accuracy of 83.59% was achieved (Additional file 1: Fig. S1). The validation accuracy for individual histology classes in the validation cohort ranged from 69.74 to 87.88%. Additionally, the classifier reached an average recall ranging from 72.0 to 90.32%, an average precision from 81.43 to 84.19%, and an average F1-score from 76.11 to 86.92% for all histology classes (Additional file 1: Fig. S2). The true histology class, the predicted histology class, and the image tile spatial coordinates with respect to each WSI were recorded for each tile. Histology classification maps were generated for visualizing spatial distribution of histological classes. Each histology class was assigned a unique color, and the predicted histology class results were spatially assembled by the image tile spatial order (Additional file 1: Fig. S3). Detailed classification results with Galway cohort can be found in Additional file 1: Figures S8–S10.

### Spatial TME features of paired histology classes predict NAC response

**Fig. 5:**
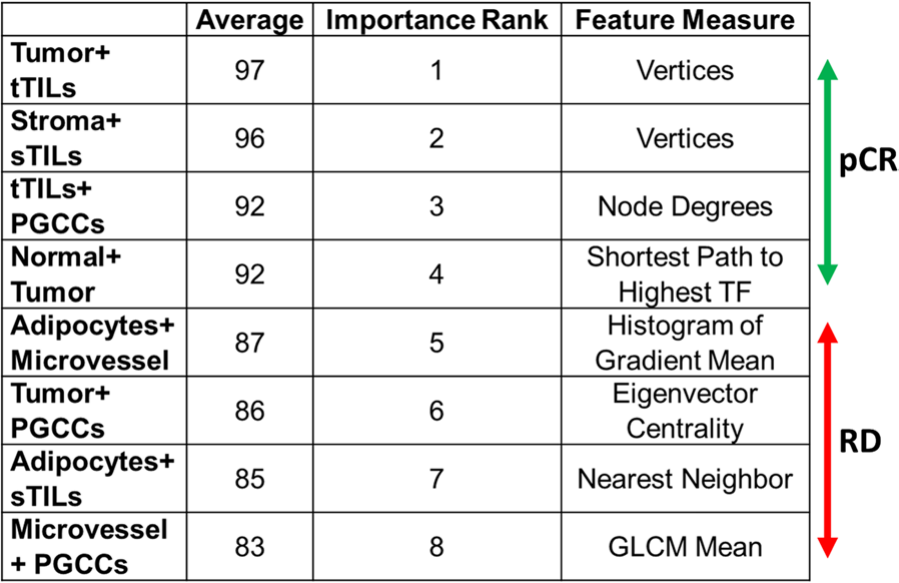
TME graph feature selection by ReliefF. TME graph features histology class pairs sorted by their importance weights from the ReliefF algorithm in the model development cohort. Abbreviations: TF, Texture Feature; sTILs, stromal TILs; tTILs, tumor TILs; PGCCs, polyploid giant cancer cells; GLCM, gray-level co-occurrence matrix

Using the importance weights from the ReliefF algorithm, we ranked all TME features from paired histology classes. For each histology class pair, we computed 20 TME features that were related to texture feature averages using a random geometric construct, local node-based features using a spectral construct, and global graph-based features using a minimum spanning tree construct (Additional file 1: Table S2). The top eight histology pairs are presented in Fig. 5. For example, the histological class pair of tumors and tTILs with the largest importance weight is known to be strongly associated with pCR. In contrast, the histological class pair of microvessel density and PGCCs strongly correlate with RD. These results are in line with previously published studies [58–66].

**Fig. 6:**
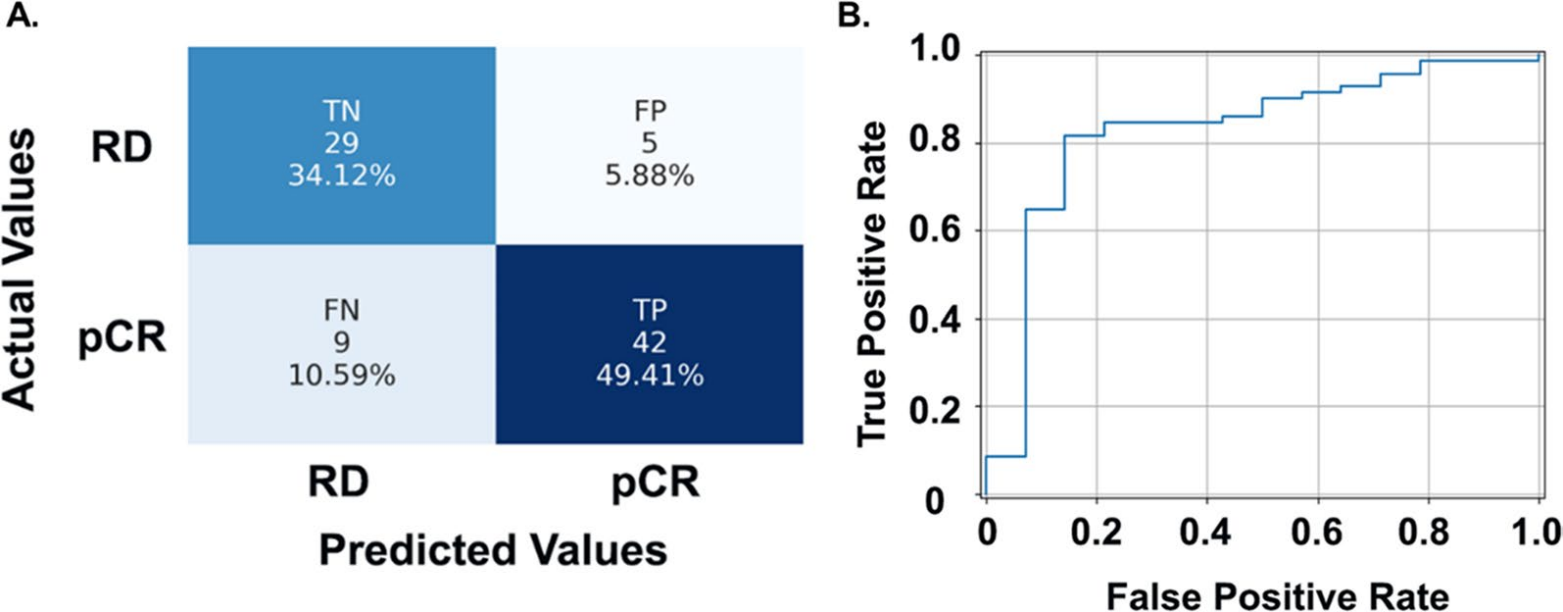
Patient-level NAC response prediction performance of the best classifier by LOO-CV in the model development cohort. **A** Confusion matrix showing performance of the rbfSVM model. **B** ROC curve of the best NAC response prediction pipeline. Abbreviations: LOO-CV, leave-one-out cross-validation; rbfSVM, radial basis function SVM; ROC, receiver operating characteristic; TN, true negative; FP, false positive; FN, false negative

With each TNBC patient represented by selected TME features, we trained and tested the classifier for NAC response prediction using LOO-CV strategy. The prediction accuracies by 1NN, linSVM, and ensemble tree were 72.90%, 62.43%, and 70.61%, respectively. Similar to the tile-level histology classification, rbfSVM achieved the best NAC response prediction at the patient level with a prediction accuracy of 83.53%. Out of 51 cases, 42 were correctly predicted as pCR. Twenty-nine out of 34 patients were correctly predicted as RD (Fig. 6A). The FP and FN groups included five and nine misclassified patients, respectively, resulting in a specificity of 85.29% and sensitivity of 82.35% (Fig. 6A). Additionally, the receiver operating characteristic (ROC) curve of the best-performing NAC prediction pipeline is presented in Fig. 6B with area under the curve (AUC) reaching 0.83. Detailed prediction performance with the Emory cohort is presented in Table 2.

**Table 2:** NAC prediction performance with Emory Hospital cohort

	Accuracy	AUC	Sensitivity	Specificity	Precision	F1
1NN	0.729 [0.703, 0.756]	0.735 [0.690, 0.779]	0.745 [0.693, 0.797]	0.706 [0.676, 0.736]	0.792 [0.755, 0.828]	0.768 [0.747, 0.788]
Linear SVM	0.624 [0.586, 0.660]	0.709 [0.654, 0.764]	0.490 [0.425, 0.555]	0.824 [0.801, 0.846]	0.806 [0.774, 0.839]	0.610 [0.581, 0.638]
RBF SVM	0.835 [0.808, 0.862]	0.827 [0.786, 0.868]	0.824 [0.768, 0.879]	0.853 [0.818, 0.887]	0.894 [0.849, 0.938]	0.857 [0.823, 0.892]
EnsembleTree RUSBoost	0.706 [0.686, 0.725]	0.681 [0.648, 0.714]	0.784 [0.735, 0.833]	0.588 [0.566, 0.611]	0.741 [0.705, 0.777]	0.762 [0.740, 0.784]

The average and 95% confidence interval of accuracy, AUC, sensitivity, specificity, precision, and F1-Score are presented for each model by a leave-one-out cross-validation strategy

The top eight graph-derived features capture histological information from the TME that is critical for NAC response prediction. Additional file 1: Figure S4 highlights representative tissue regions from where image tiles of the associated paired histology classes were derived for TME graph nodes. The resulting attention maps are tissue areas with high discriminating values for NAC response prediction, i.e., pCR vs RD (Additional file 1: Fig. S4).

### Validating the NAC response prediction performance in an independent cohort

**Fig. 7:**
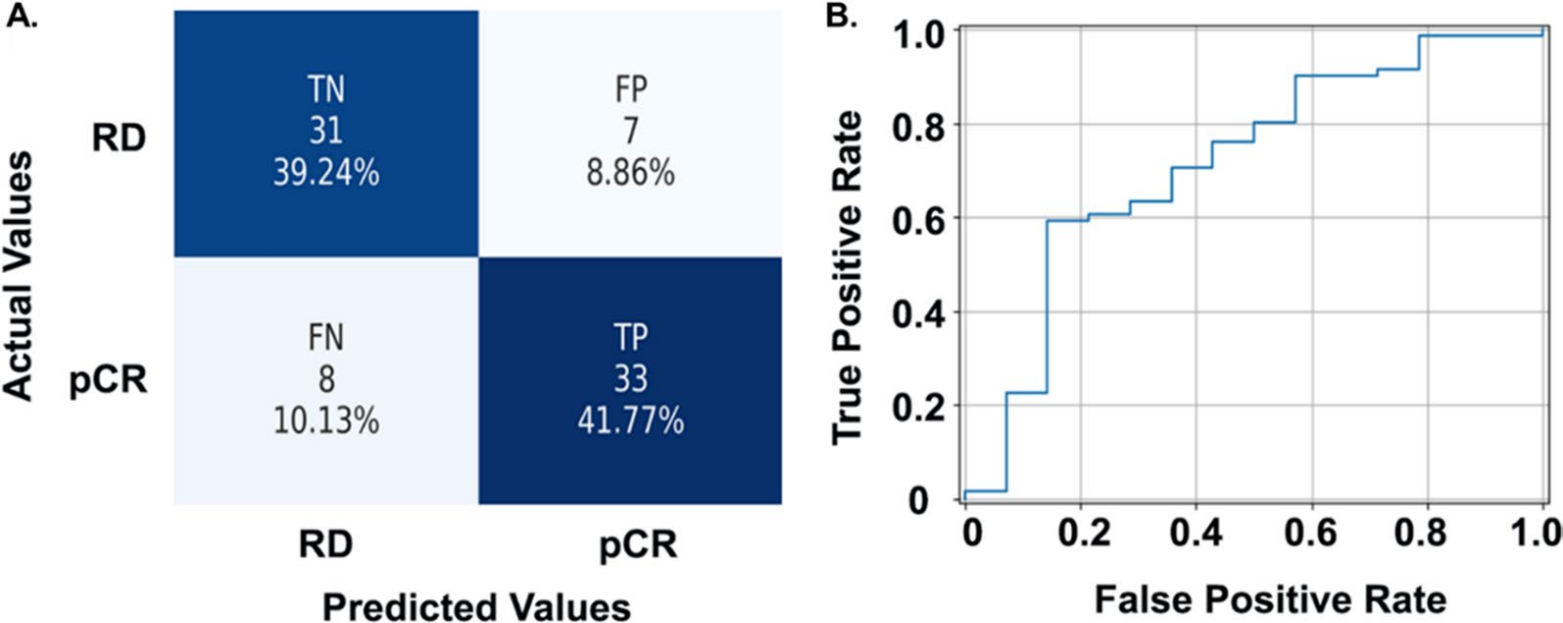
The NAC prediction model's performance in the validation cohort. **A** Confusion matrix showing performance of the best NAC response prediction model. **B** ROC curve of the best NAC response prediction pipeline. Abbreviations: LOO-CV, leave-one-out cross-validation; rbfSVM, radial basis function SVM; ROC, receiver operating characteristic; TN, true negative; FP, false positive; FN, false negative; TP, true positive

We validated the prediction performance of the pipeline in an independent cohort consisting of 79 WSIs. Using the same image quality checks and preprocessing steps, we analyzed each WSI using the previously trained classifiers. The positive and negative group included 41 and 38 patients, respectively. Our prediction method correctly predicted 33 and 31 patients from the positive and negative groups, respectively, with a prediction accuracy of 81.01%. The FP and FN groups included seven and eight misclassified patients, respectively, resulting in a specificity of 81.58% and sensitivity of 80.49% (Fig. 7A). Additionally, the ROC curve of the best NAC prediction pipeline is presented in Fig. 7B, with AUC reaching 0.83. Detailed prediction performance with the Galway cohort is presented in Table 3.

**Table 3:** NAC prediction performance with Galway cohort

	Accuracy	AUC	Sensitivity	Specificity	Precision	F1
1NN	0.684 [0.645, 0.717]	0.671 [0.524, 0.824]	0.585 [0.522, 0.648]	0.789 [0.765, 0.814]	0.75 [0.712, 0.788]	0.653 [0.619, 0.696]
Linear SVM	0.608 [0.572, 0.643]	0.617 [0.574, 0.660]	0.488 [0.428, 0.548]	0.737 [0.707, 0.766]	0.667 [0.643, 0.690]	0.563 [0.542, 0.585]
RBF SVM	0.810 [0.783, 0.837]	0.832 [0.792, 0.873]	0.805 [0.749, 0.860]	0.816 [0.779, 0.853]	0.825 [0.787, 0.863]	0.815 [0.786, 0.844]
EnsembleTree RUSBoost	0.671 [0.643, 0.699]	0.691 [0.654, 0.727]	0.683 [0.625, 0.741]	0.658 [0.622, 0.694]	0.683 [0.645, 0.721]	0.683 [0.652, 0.714]

The average and 95% confidence interval of accuracy, AUC, sensitivity, specificity, precision, and F1-Score are presented for each model by a leave-one-out cross-validation strategy

## Discussion

Women with TNBC exhibit significantly worse 5-year survival rates than those with non-TNBC, regardless of the tumor stage at diagnosis [69]. No targeted or endocrine therapy is available for TNBC, and NAC is the cornerstone of treatment. However, only 30–40% of TNBC patients achieve pCR with NAC, and there is a dire need for early identification of the nearly 70% of patients who should be offered alternative regimens to improve treatment outcomes. In this study, we used ML approaches to predict NAC response and stratify patients into NAC responders and non-responders based on H&E-stained WSIs of tissue biopsies. We developed a two-step prediction model: in the first step, the histology class of each H&E image tile was determined using a tile-level classification pipeline; in the second step, the spatial graph-derived features associated with histology class pairs were used to predict patient-level NAC response (pCR vs RD). Our model unveils and leverages novel NAC response predictive features and spatial patterns of TME histology components from WSIs of TNBC tissue biopsies. This study also highlights the role of various TME components in accurately predicting NAC response.

TME components and their interactions can influence NAC response in patients with TNBC [70–72]. Traditional methods using human annotations are unable to capture these spatial relationships. In contrast, our approach incorporates the spatial relationships of various TME components to predict NAC response in patients with TNBC. Using a graph structure for spatial TME characterization, we identified eight histology component pairs that accurately predicted NAC response. We expect that an investigation with higher-order combinations (e.g., tertiary and quaternary) can further increase NAC response prediction accuracy. The top three TME features captured the spatial interactions between (1) tumor cells and tTILs, (2) stroma and sTILs, and (3) tTILs and PGCCs. Studies have shown the predictive importance of tumor area, immune activation markers, and TILs in TNBC biopsies [73–76]. Our results provide further evidence that the interrelationships between TILs, stroma, adipocytes, and tumor cells can predict NAC response in patients with TNBC. Other recently published studies that have relied on WSI models [77, 78] include one that used a federated learning model to predict NAC response in TNBC, and found hemorrhage, TILs, and necrosis as predictive of pCR and apocrine change, fibrosis, and noncohesive tumor cells being predictive of RD [77]. Another study quantified the stromal and tumor features in a WSI-based multi-omic (WSI, clinical, pathological) ML model and found that high collagenous stroma was best associated with lower pCR rates [78]. Our study used expert annotations that effectively guided the ML models to identify specific histological patterns in spatial TME contexts. While our supervised ML model identified the common histological component of TILs, it did not rank hemorrhage, necrosis, fibrosis, and apocrine change as important predictors due to the lack of annotated training data.

Our NAC response prediction pipeline provides classification accuracy and attention maps that can be highly useful in clinical practice. Attention maps help pathologists and researchers by identifying tissue regions in a WSI that are highly predictive of NAC response, thereby improving slide review, reducing visual fatigue, and facilitating image data interpretation. Information from attention maps can be combined with other WSI-derived data such as, Ki67 and pH3 immunohistochemistry-stained serial tissue sections, to train deep-learning models for enhanced prediction [79–81]. Ki67 and pH3 are clinical biomarkers with demonstrated NAC response predictive value in TNBC tumors [82, 83]. Furthermore, our predictive model is promising for integrating data from various sources, such as electronic health records, laboratory test results, and demographic information, to provide predictions based on the overall view of the health status of patients.

Limitations of the study include small sample sizes, slide quality issues, and expensive computational processes. Quality checks are necessary to ensure inclusion of adequate samples to develop effective training classifiers. The different slide staining protocols, artifacts, and plating variances from different institutions (e.g., cutting glass slide edges) may have resulted in inconsistencies in slide quality. Thus, although we had a larger number of WSIs to begin with, the final validation cohort was whittled down. Because the sample size was small, there was an imbalance of histology classes presented among different patient slides. More histology classes (e.g., microcalcification, muscle) should be included to improve the training of the tile-level histology classifier in all histology classes. We had two pathologists independently annotate the WSIs; however, more experts can be included in the future to validate the annotations and reduce interobserver variability. Additionally, our pipeline is computationally expensive because multiple processes occur throughout the pipeline such as partitioning gigapixel WSIs, calculating various feature measures for each tile, constructing graphs based on spatial relationships. Computational constraints can stem from institutional high-performance computing (HPC) server data loss, standard maintenance, and outages. Refining the code for faster processing times (parallel processing) based on an advanced computer architecture could help support ML processes and data management. We cannot identify important spatially related histological features using image viewing software alone because the software is not scalable for large datasets. Each digital pathology software is limited in the amount of data processed through its graphical user interfaces before exceeding the computational capabilities.

Future work will include model validation in a larger cohort. Future work will also include the development of prediction models with higher-order feature combinations and graph convolution networks. It is important to develop an efficient pipeline to increase the amount of image data and decrease the computational time. Additionally, combinations of features with the highest predictive value will be used to increase the predictive power of the full feature set. For example, attention map regions can be leveraged to focus on regions of interest, which can be used for more complex analyses, such as imaging mass cytometry, to distinguish between the various TIL subtypes and to further refine NAC response prediction. We also plan to extend our pipeline to incorporate other tissue stains, including immunohistochemistry. A more efficient pipeline can reduce the frequency of false negatives and thus minimize the risk of undertreating patients, which can result in early relapse and poor outcomes.

## Conclusions

Using feature engineering and supervised ML, we demonstrated the strong discriminating power of TME histological components and their spatial relationships in predicting NAC response in patients with TNBC. Among 120 histology feature pairs, we identified eight with the highest predictive value. The most predictive histology feature pair for pCR was tumor and tTILs, whereas microvessel density and PGCCs was the feature pair most strongly correlated with RD. The proposed ML pipeline can help identify tissue areas in H&E-stained WSIs with a high predictive value for NAC response prediction and can help in clinical decision-making.

### Supplementary Information

**Additional file 1:** The supplementary files include Figures S1-S10 of results, Tables S1 and S2 of the feature list, Methods S1 and S2 of feature extraction, and the supplementary file’s bibliography.

## Data Availability

The data underlying this article will be shared on request to the corresponding author.
