# Supplementary material for: Digital image analysis and machine learning-assisted prediction of neoadjuvant chemotherapy response in triple-negative breast cancer
Source: Breast Cancer Res. 2024 Jan 18;26:12. doi: 10.1186/s13058-023-01752-y (PMC10797728; doi:10.1186/s13058-023-01752-y)
Supplement: Supplementary file 1 — Additional file 1: The supplementary files include Figures S1-S10 of results, Tables S1 and S2 of the feature list, Methods S1 and S2 of feature extraction, and the supplementary file’s bibliography. [file 13058_2023_1752_MOESM1_ESM.docx]

**Supplementary Files**


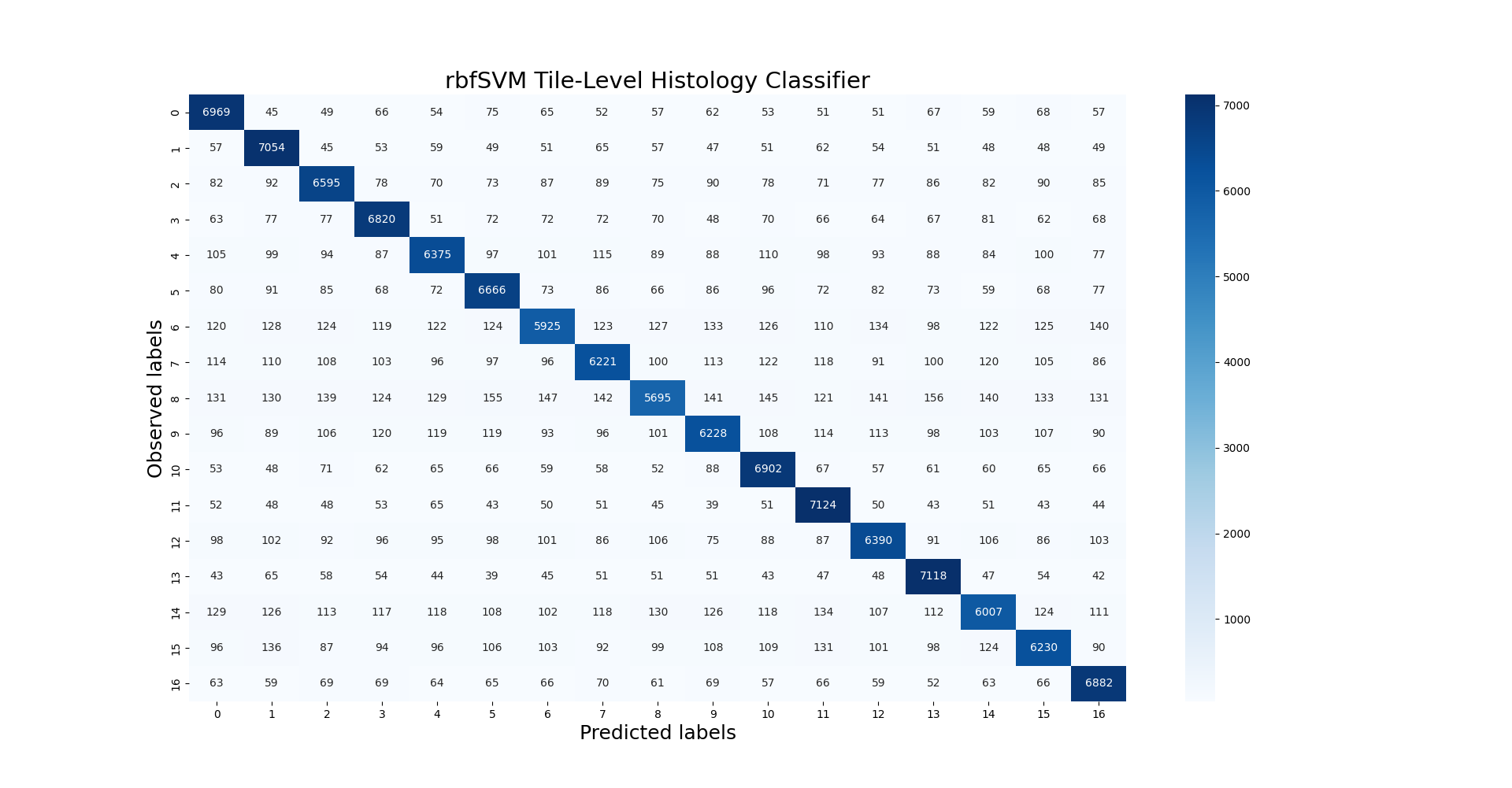


**Figure S1. Testing Tile-level histology classification performance in the validation cohort.** Confusion matrix showing the performance of the rbfSVM model for tile-level histology class prediction (0, stroma; 1, tumor; 2, tertiary TILs; 3, stromal TILs; 4, normal tissue; 5, polyploid giant cancer cells; 6, veins; 7, necrosis; 8, microvessels; 9, benign tumor; 10, tumor TILs; 11, in situ carcinoma; 12, hemorrhage; 13, adipocytes; 14, apocrine change; 15, mucinous change; 16, background). The diagonal elements (true positives shown in dark blue) divided by the total number of samples in a row (light blue) give the accuracy of the histology class. The background class was not used in the interpretation of results.


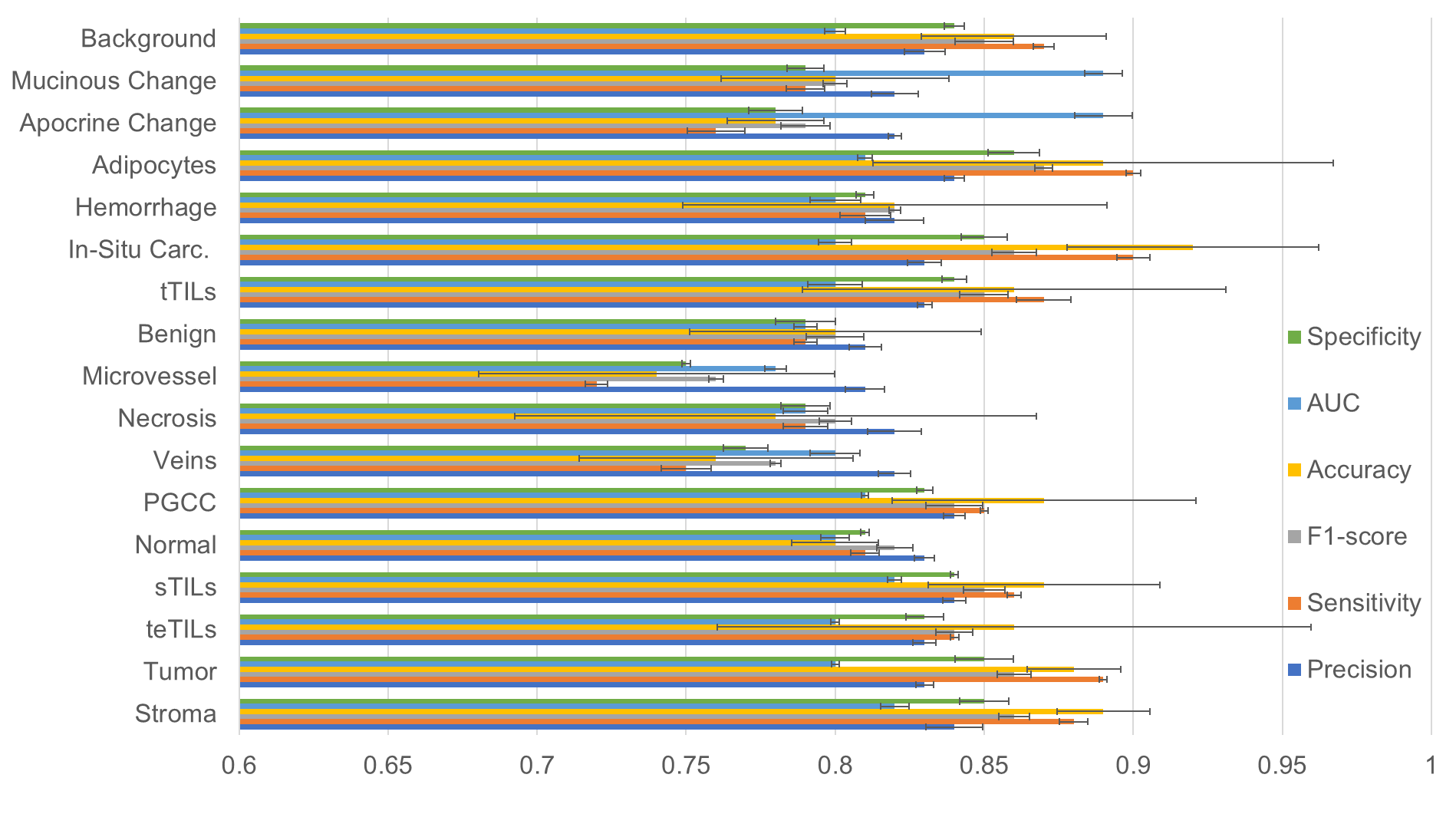
 **Figure S2.** Testing tile-level histology classification performance of the rbfSVM classifier with the Galway validation cohort. Each bar represents the weighted average of tiles and their predicted probabilities during testing for a histology class. Specificity (green) measures how often the rbfSVM classifier correctly predicted true negatives. AUC (blue) reflects the model's ability to distinguish between positive and negative classes. Accuracy (yellow) indicates the proportion of correct predictions out of total predictions. F1-score (grey) presents a balanced view of rbfSVM classifier performance. Sensitivity (orange) suggests how often the rbfSVM classifier correctly identifies positive instances. Precision (blue) indicates how often the rbfSVM classifier correctly predicts true positives. Error bars represent the 95% confidence interval in each case.


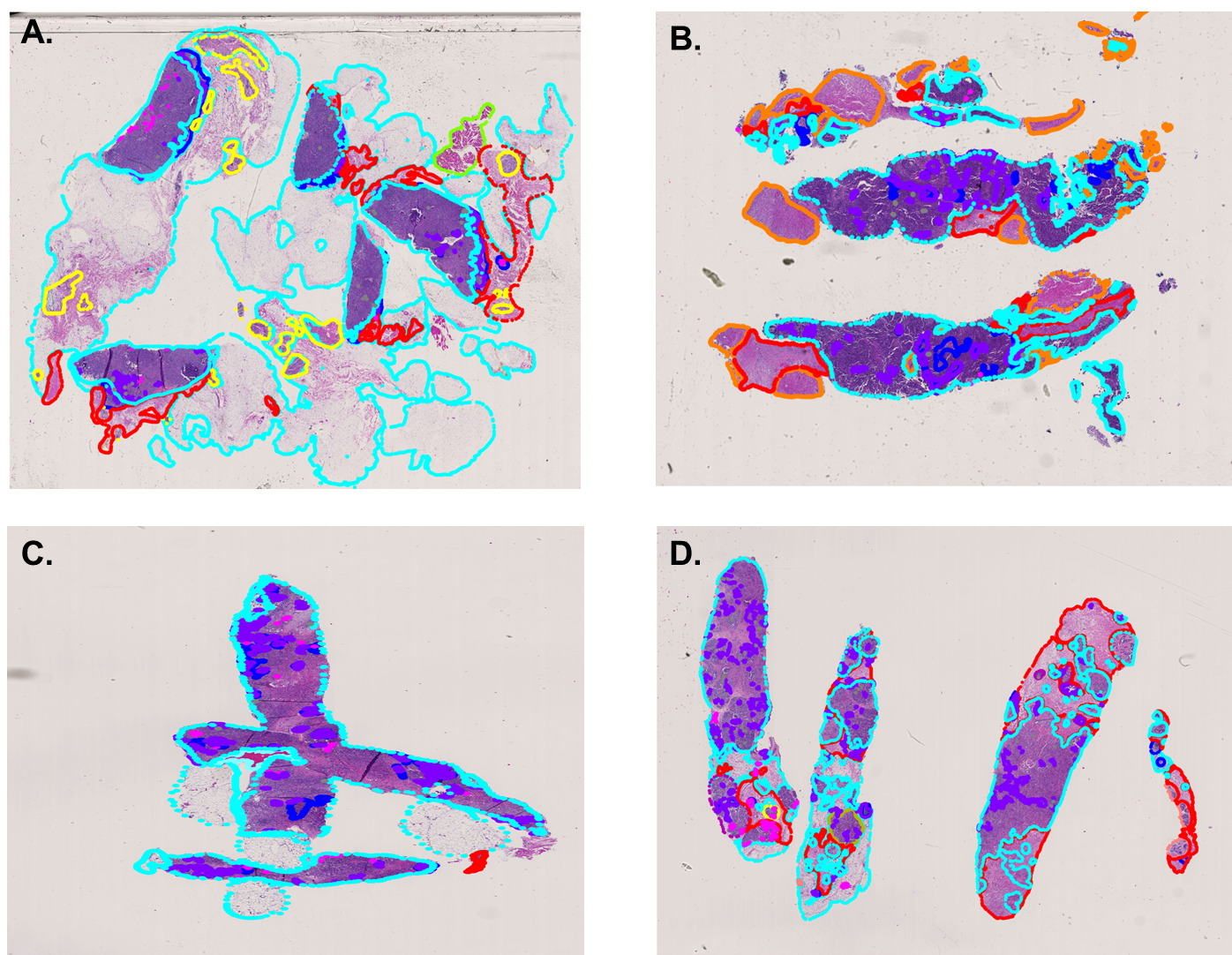


**Figure S3. Representative classification maps.** (A-B) Two pCR cases and (C-D) two RD cases are presented with color coded histology labels. A & C are annotated WSIs. B & D are with predicted labels. The color codes for each label is as follows: stroma (red), blood vessel (blue), MVD (green), Adipose tissue (cyan), PGCC (magenta), Normal breast tissue (yellow), Necrosis (orange), StromaTILs (purple), TumorTILs (grey), Tumor (aqua), tertiaryLS (fuchsia), mucinous change (maroon), in-situ (olive), hemorrhage (teal), benign breast tumor (salmon), apocrine change (indigo). All labels were mapped to a WSI, even if they were removed from the feature extraction portion of the pipeline.


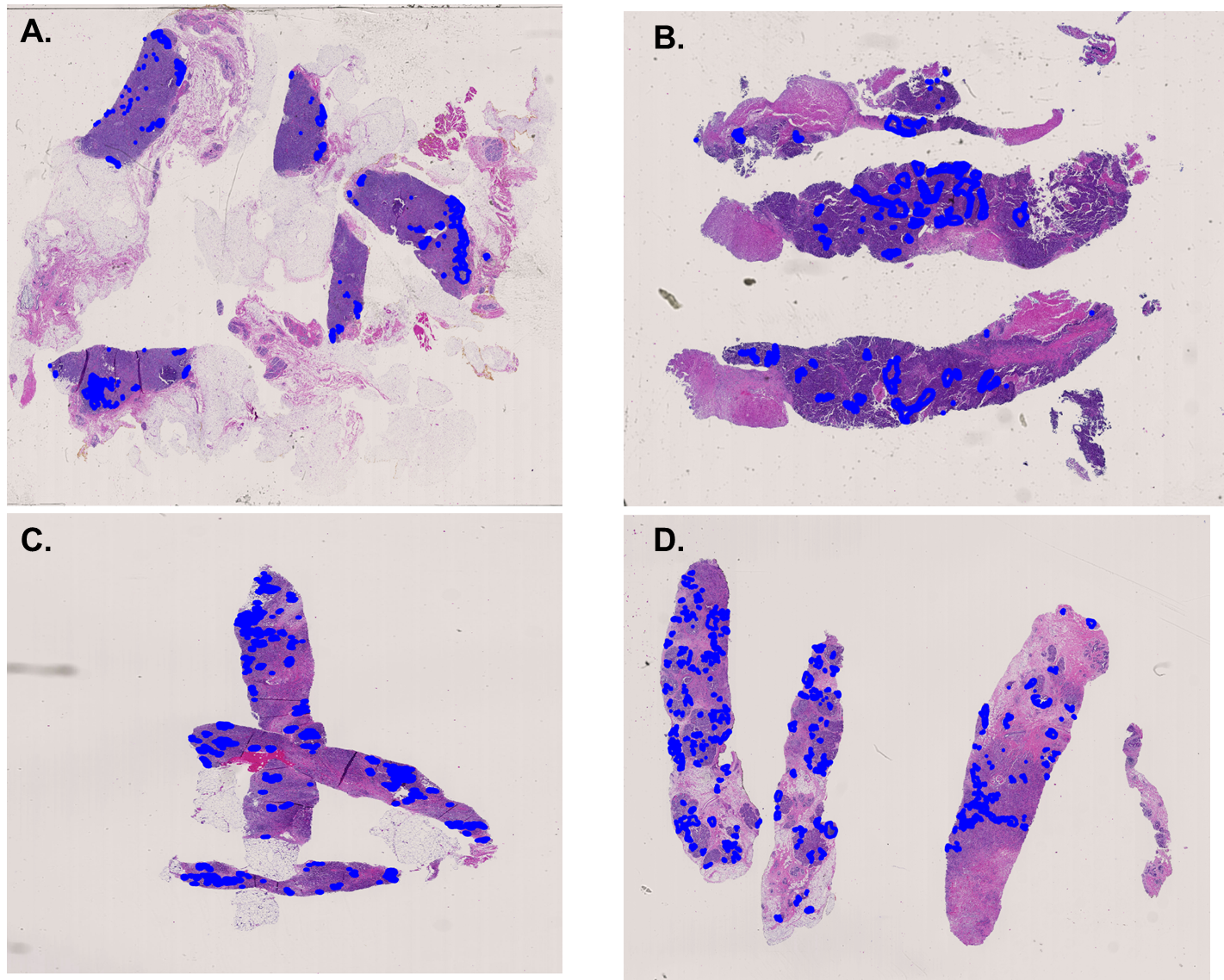


**Figure S4. Representative attention maps.** Attention maps of cases in Fig. S3 are presented with the full graph-feature set (Table S1 and S3). Each feature is highlighted in blue. Each tile size is 224 x 224 and is in reference to the region of interest of the full graph-feature set.


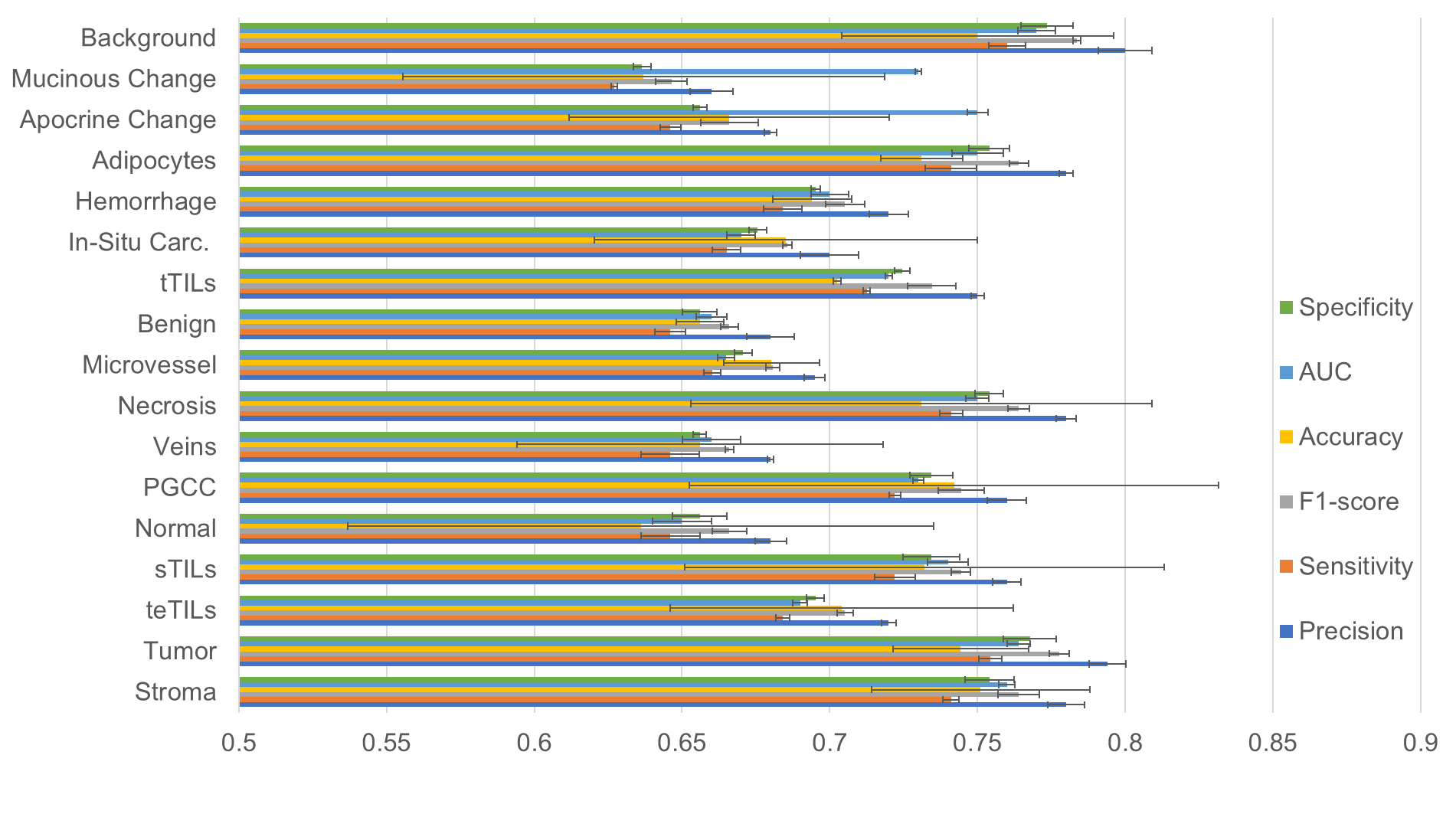


**Figure S5. Testing tile-level histology classification performance of the ensemble RUSBoost classifier with the Emory Hospital development cohort.** Each bar represents the weighted average of tiles and their predicted probabilities during testing for a histology class. Specificity (green) measures how often the ensemble RUSBoost classifier correctly predicted true negatives. AUC (blue) reflects the model's ability to distinguish between positive and negative classes. Accuracy (yellow) indicates the proportion of correct predictions out of total predictions. F1-score (grey) presents a balanced view of ensemble RUSBoost classifier performance. Sensitivity (orange) suggests how often the ensemble RUSBoost classifier correctly identifies positive instances. Precision (blue) indicates how often the ensemble RUSBoost classifier correctly predicts true positives. Error bars represent the 95% confidence interval in each case.


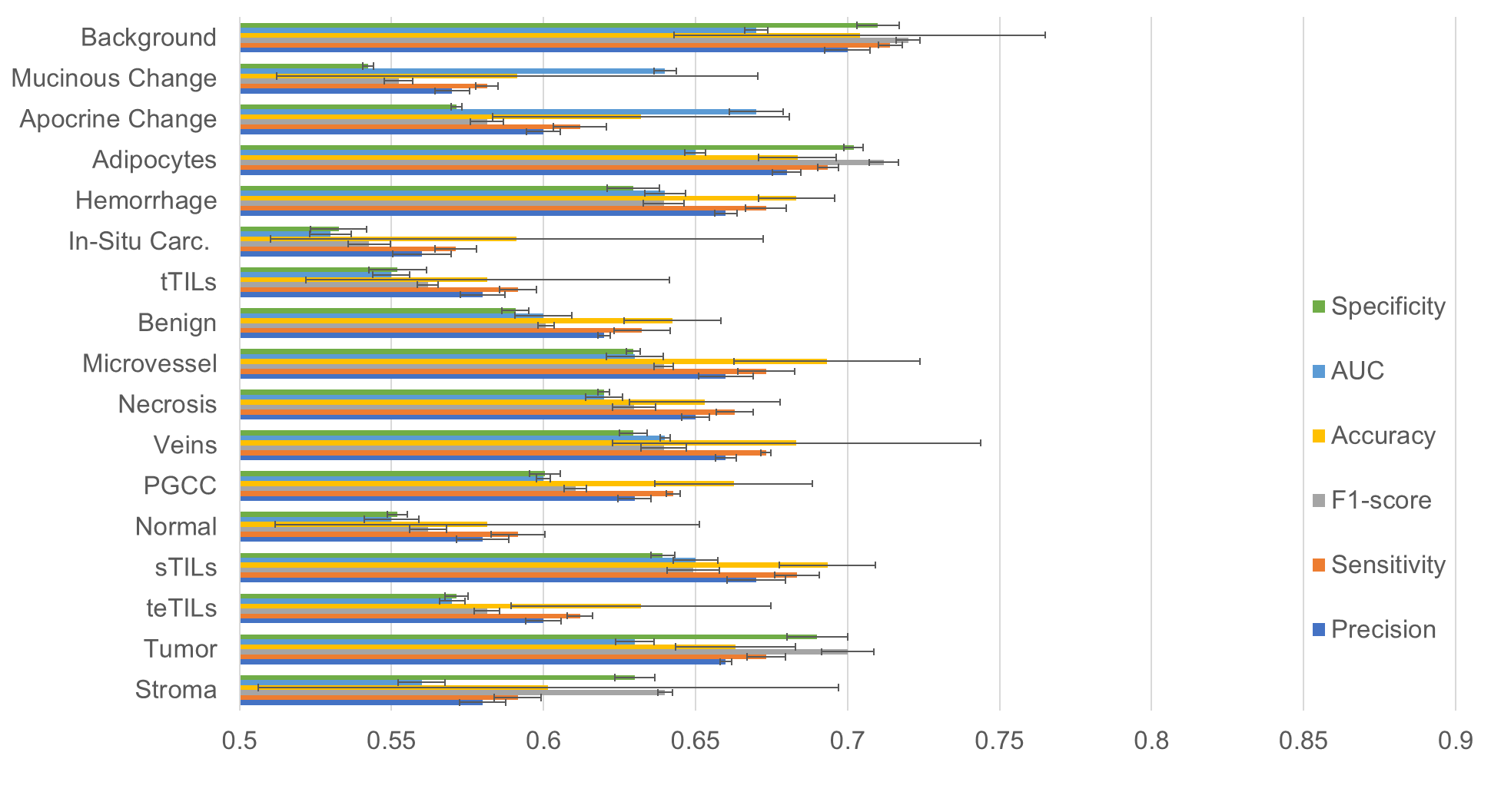


**Figure S6. Testing tile-level histology classification performance of the linSVM classifier with the Emory Hospital development cohort**. Each bar represents the weighted average of tiles and their predicted probabilities during testing for a histology class. Specificity (green) measures how often the linSVM classifier correctly predicted true negatives. AUC (blue) reflects the model’s ability to distinguish between positive and negative classes. Accuracy (yellow) indicates the proportion of correct predictions out of total predictions. F1-score (grey) presents a balanced view of linSVM classifier performance. Sensitivity (orange) suggests how often the linSVM classifier correctly identifies positive instances. Precision (blue) indicates how often the linSVM classifier correctly predicts true positives. Error bars represent the 95% confidence interval in each case.


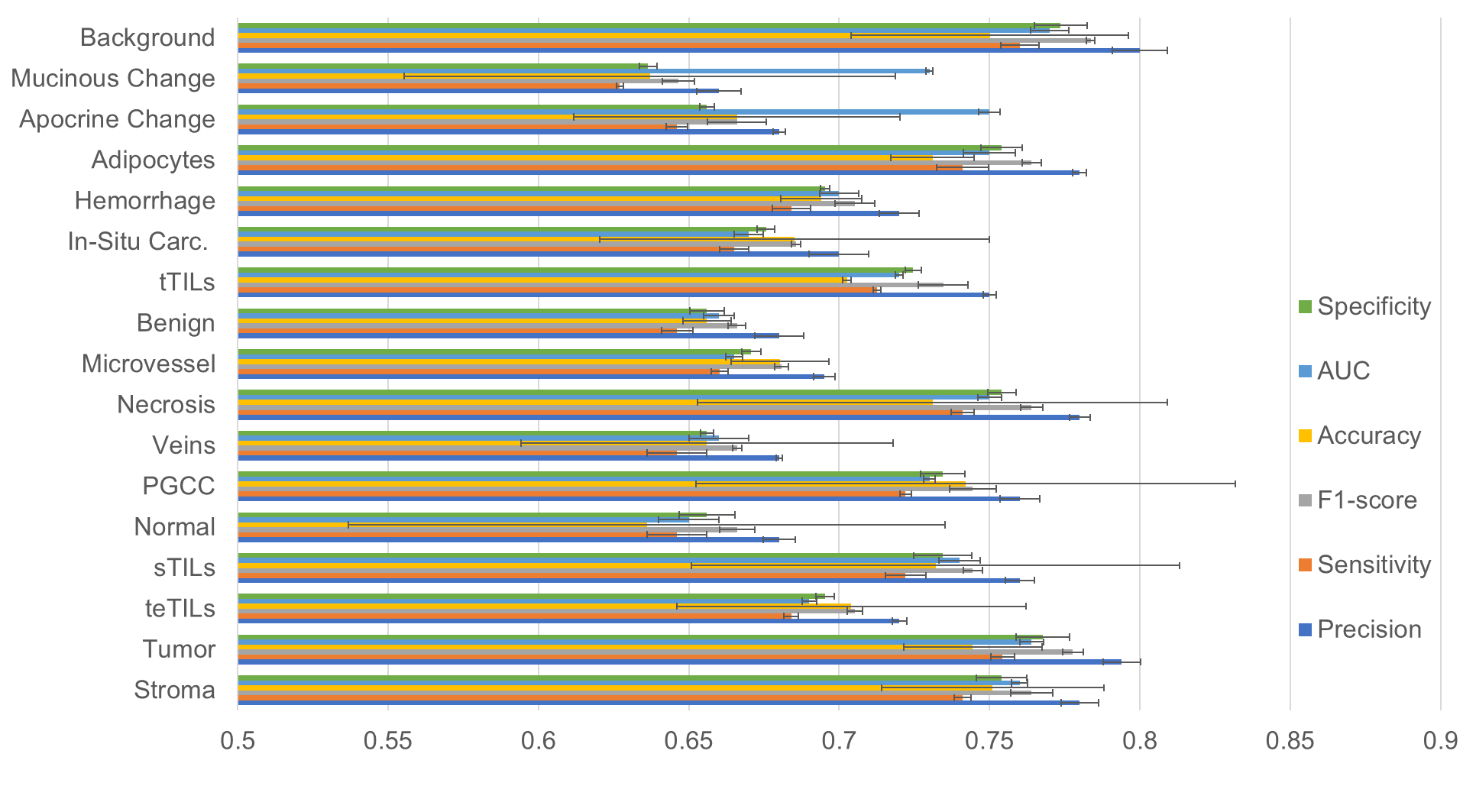


**Figure S7. Testing tile-level histology classification performance of the 1NN classifier with the Emory Hospital development cohort**. Each bar represents the weighted average of tiles and their predicted probabilities during testing for a histology class. Specificity (green) measures how often the 1NN classifier correctly predicted true negatives. AUC (blue) reflects the model's ability to distinguish between positive and negative classes. Accuracy (yellow) indicates the proportion of correct predictions out of total predictions. F1-score (grey) presents a balanced view of 1NN classifier performance. Sensitivity (orange) suggests how often the 1NN classifier correctly identifies positive instances. Precision (blue) indicates how often the 1NN classifier correctly predicts true positives. Error bars represent the 95% confidence interval in each case.


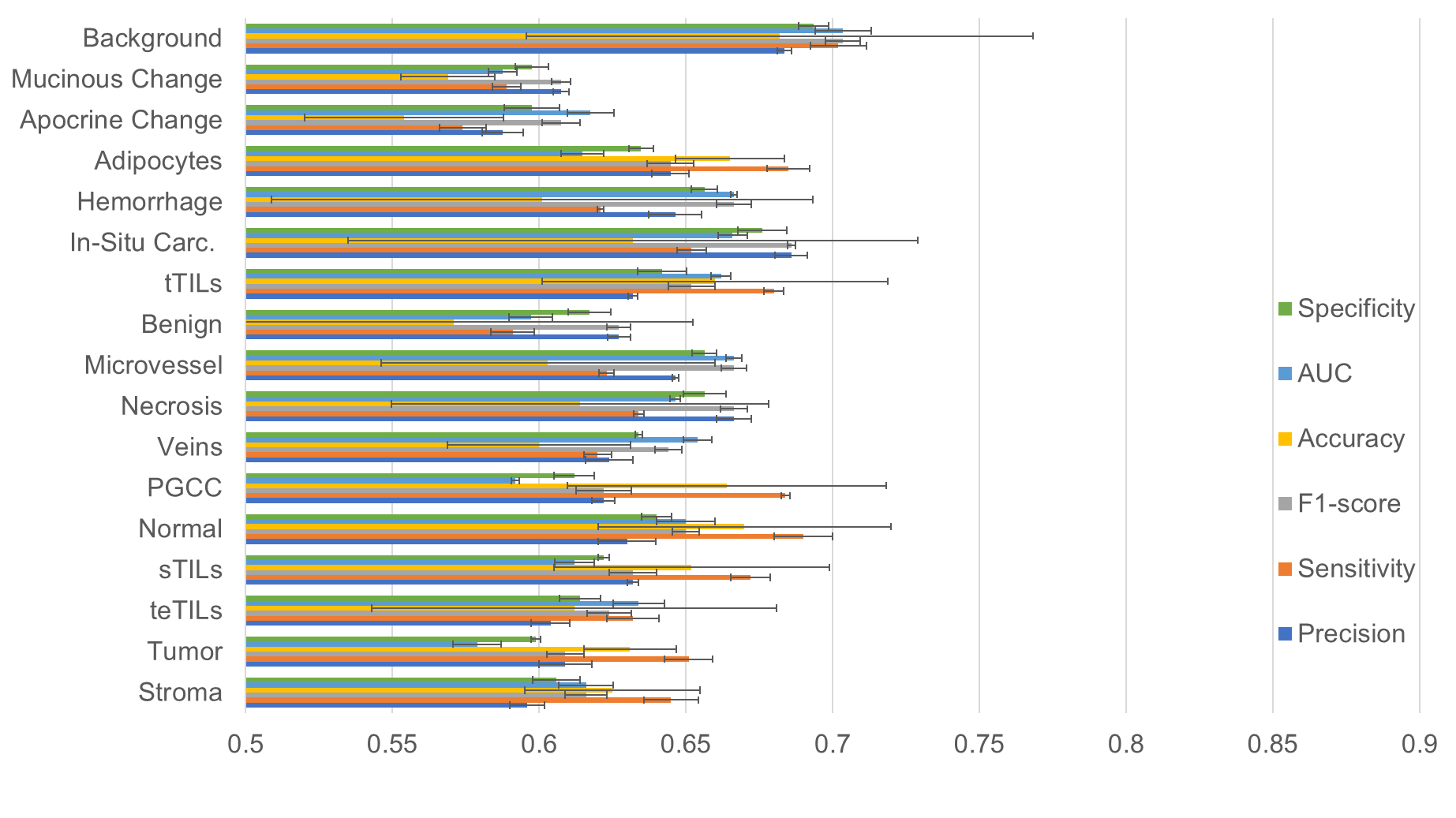


**Figure S8. Testing tile-level histology classification performance of the ensemble RUSBoost classifier with the Galway validation cohort**. Each bar represents the weighted average of tiles and their predicted probabilities during testing for a histology class. Specificity (green) measures how often the ensemble RUSBoost classifier correctly predicted true negatives. AUC (blue) reflects the model's ability to distinguish between positive and negative classes. Accuracy (yellow) indicates the proportion of correct predictions out of total predictions. F1-score (grey) presents a balanced view of ensemble RUSBoost classifier performance. Sensitivity (orange) suggests how often the ensemble RUSBoost classifier correctly identifies positive instances. Precision (blue) indicates how often the ensemble RUSBoost classifier correctly predicts true positives. Error bars represent the 95% confidence interval in each case.


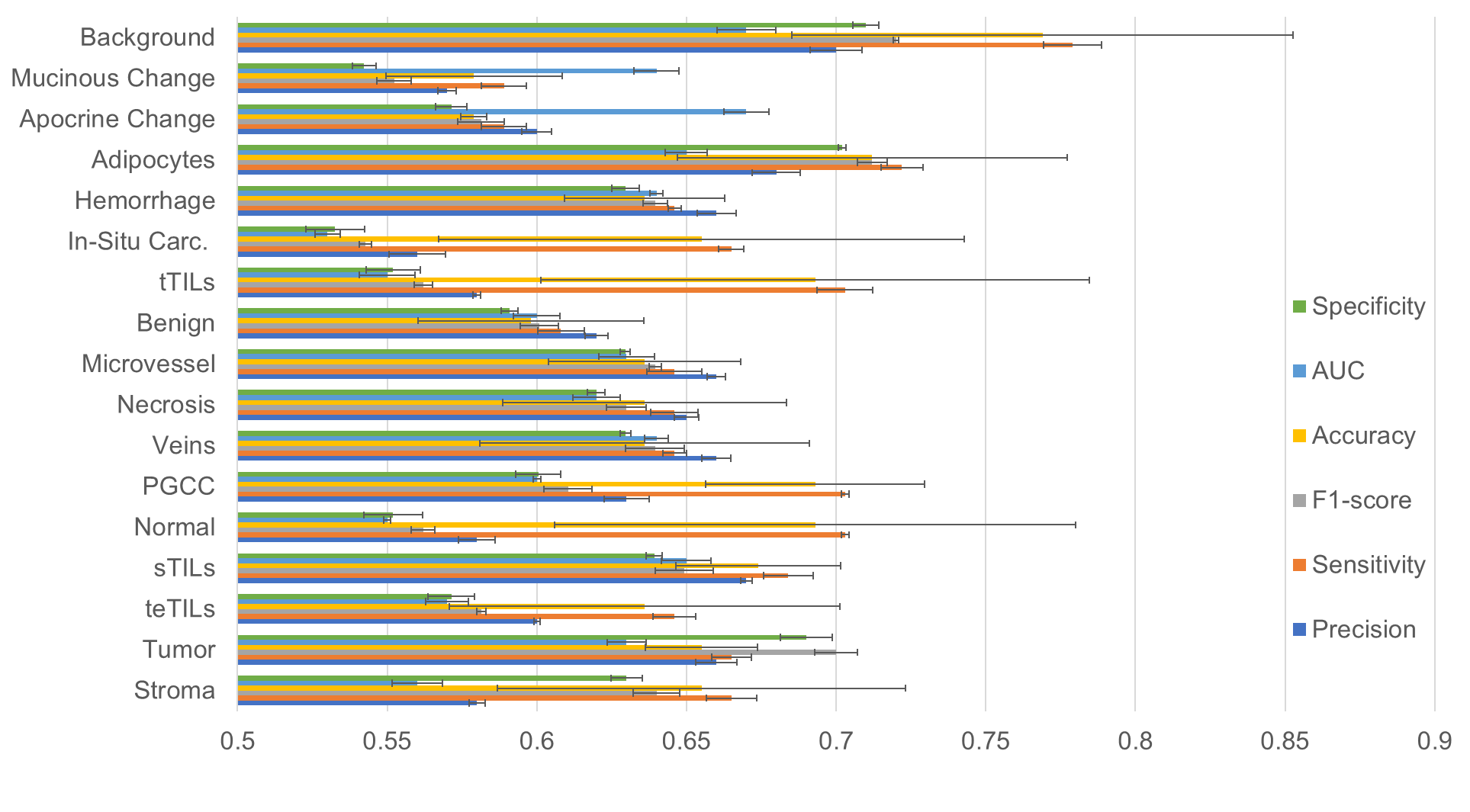


**Figure S9. Testing tile-level histology classification performance of the linSVM classifier with the Galway validation cohort**. Each bar represents the weighted average of tiles and their predicted probabilities during testing for a histology class. Specificity (green) measures how often the linSVM classifier correctly predicted true negatives. AUC (blue) reflects the model's ability to distinguish between positive and negative classes. Accuracy (yellow) indicates the proportion of correct predictions out of total predictions. F1-score (grey) presents a balanced view of linSVM classifier performance. Sensitivity (orange) suggests how often the linSVM classifier correctly identifies positive instances. Precision (blue) indicates how often the linSVM classifier correctly predicts true positives. Error bars represent the 95% confidence interval in each case.


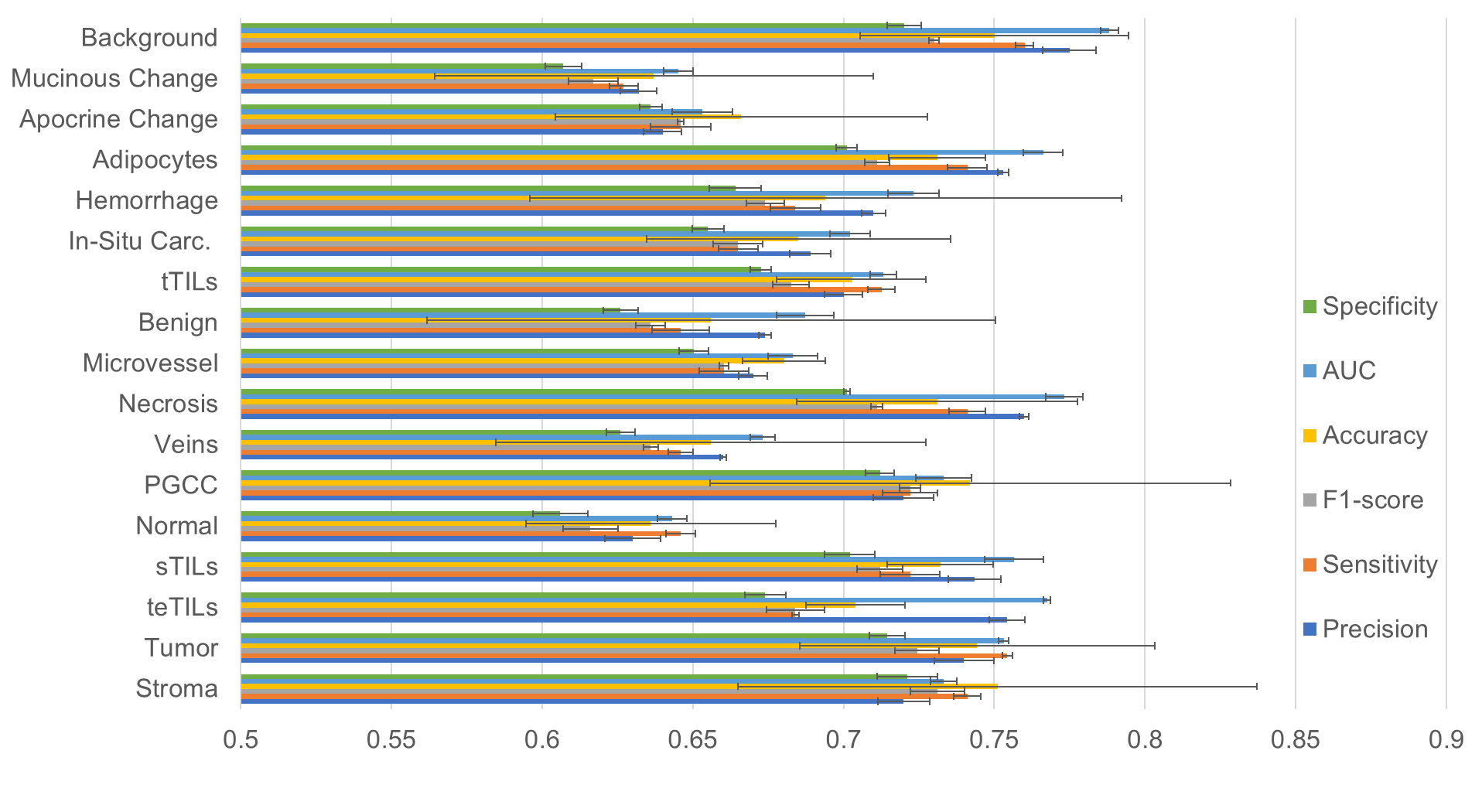


**Figure S10. Testing tile-level histology classification performance of the 1NN classifier with the Galway validation cohort**. Each bar represents the weighted average of tiles and their predicted probabilities during testing for a histology class. Specificity (green) measures how often the 1NN classifier correctly predicted true negatives. AUC (blue) reflects the model's ability to distinguish between positive and negative classes. Accuracy (yellow) indicates the proportion of correct predictions out of total predictions. F1-score (grey) presents a balanced view of 1NN classifier performance. Sensitivity (orange) suggests how often the 1NN classifier correctly identifies positive instances. Precision (blue) indicates how often the 1NN classifier correctly predicts true positives. Error bars represent the 95% confidence interval in each case.

**Table S1 Texture features for tile representation.** Texture features were generated using six feature extraction methods to represent each image tile [supplementary methods S1(A-E)].

| **Texture Feature** | **Feature Number** | **Retained Feature #** | **Source** |
| --- | --- | --- | --- |
| Gray-Level Concurrent Matrix (GLCM) | 20 | 0 | (1) |
| Gabor Filter | 6 | 6 | (2-4) |
| Local Binary Patterns (LBP) | 34 | 34 | (5, 6) |
| Tamura | 5 | 0 | (7) |
| Lower-Order Histogram | 5 | 5 | (8, 9) |
| Higher-Order Histogram | 10 | 10 | (8, 9) |

**Table S2.** **TME features for patient representation.** Each patient tile was represented by 20 TME features related to texture feature means, local node-based features, and

global graph-based features. [supplementary methods S2(A-D)].

| **Spatial TME Feature** | **Feature Number** | **Source** |
| --- | --- | --- |
| Texture Feature Average | 6 | (10) |
| Local Node | 6 | (11, 12) |
| Global Connectivity | 8 | (11, 12) |

**Method S1 – Texture feature extraction methods**

1. **Gray-level co-occurrence matrix (GLCM)** features consist of pairs of pixels in certain spatial relations to each other. In this study, we used displacement vectors (1–5 pixels) and the average of all four directions (0°, 45°, 90°, 135°) to make the GLCM features invariant. Four global statistics are used for GLCM feature computation, including the co-occurrence matrix energy, homogeneity, contrast, and correlation. In total, we generated 20 GLCM features per image tile.
2. **Gabor filter features** are Gaussian kernel functions modulated by a sinusoidal plane wave capable of multiresolution signal decomposition. We computed the mean intensity of the Gabor-transformed magnitude images in six directions (0°, 30°, 60°, 90°, 120°, 150°) and six wavelengths (2, 4, 6, 8, 10, and 12 pixels/cycle). To keep the features invariant, we averaged the results from all directions, reducing them to six features per image tile.
3. **Local binary pattern (LBP) features** are associated with the probability of binary patterns that occur within local structures (neighbors) of that image pixel. In this study, we used 34 rotationally invariant Fourier features (13, 14).
4. **Tamura features** correspond to visual perception based on texture descriptors. We used texture features on coarseness, contrast, directionality, line-likeness, and roughness.
5. **Histograms of gradient magnitude feature** consist of both lower- and higher-order histograms. First, a histogram of the magnitudes of the gradients is computed, and the orientation of the local gradients of pixel intensities is ignored (15). The gradient of magnitude measures the edge strength based on the difference between pixels in the neighborhood (15). Lower-order moments consist of kurtosis, variance, average, the first central moment of a histogram, and skewness totaling five features. Higher order consists of central moments from the order of 2–11. In total, 15 features (five lower, ten higher) were extracted using a histogram of gradient magnitude for each image tile.

**Method S2 – Graph-based feature extraction methods**

1. Texture feature averages (random geometric graph) include:
2. GLCM average
3. Gabor average
4. LBP average
5. Tamura average
6. Histogram of gradient (low) average
7. Histogram of gradient (high) average

Inputs:

- An H&E image represented as a 3D array (i.e., a matrix with dimensions $M\times N\times3$, where M and N are the dimensions of the image and 3 represents the RGB channels)
- A distance metric for measuring the similarity between pixels (e.g., Euclidean distance)
- The distance threshold determines the maximum distance between two pixels for them to be connected by an edge in the graph. Threshold is equal to tile size of 224 × 224 pixels.

Outputs:

- A random geometric graph is represented as a sparse matrix
- A set of node features (e.g., combined texture) computed from the H&E image

Summary: A random geometric graph is constructed by connecting pairs of nodes that are within a certain distance from each other. The distance threshold can be specified as a parameter and controls the sparsity of the resulting graph. Random geometric graphs help capture the geometric structure of the data and can be used to identify clusters or other patterns in the data. Total feature count for texture feature WSI average based on this calculation of texture feature per paired classification map.

1. Local node-based (spectral graph) features include:
2. Node degree
3. Eigenvector centrality
4. Clustering coefficient
5. Average number of neighboring nodes
6. Neighbor of nodes
7. Nearest neighbors

Inputs:

- An H&E image is represented as a 3D array
- The number of clusters to partition the graph into (i.e., the number of distinct tissue types in the image)

Outputs:

- A spectral graph is represented as a sparse matrix
- A set of node features (e.g., combined texture) computed from the H&E image
- A partition of the graph into the specified number of clusters

Summary: Spectral clustering uses a Laplacian graph matrix to partition the graph into clusters based on the eigenvalues and eigenvectors of the matrix. The Laplacian matrix is computed from the similarity matrix, which can be constructed using Euclidean distance. Spectral graphs help partition the data into clusters based on the graph structure and can be used to identify distinctive regions in the image.

1. Global graph-based (minimum spanning tree) features include:
2. Shortest path to highest texture feature tile histological region
3. Shortest path to lowest texture feature tile histological region
4. Closeness centrality
5. The eccentricity of a node – maximum value of the shortest path from a given node to any other node
6. Average eccentricity
7. Number of vertices
8. Trace of adjacency – sum of the eigenvalues of the adjacency matrix
9. The energy of adjacency – square sum of trace

Inputs:

- An H&E image is represented as a 3D array
- A distance metric for measuring the similarity between pixels by Euclidean distance

Outputs:

- A minimum spanning tree (MST) is represented as a sparse matrix
- A set of node features (e.g., combined texture) computed from the H&E image

Summary: MST construction involved computing a pairwise distance matrix between all pairs of nodes in the graph and then finding the tree that spans all the nodes with the minimum total edge weight. The edge weight is typically a function of the distance metric, with closer pixels having a lower weight than more distant pixels. The node features were augmented with spatial coordinates computed using dimensionality reduction techniques (principal component analysis, PCA) to retain the spatial information of the graph centroids. The tree structure helped encode the spatial relationships between the data points retained for NAC response prediction.

D. Graph-based WSI features are used to determine the NAC response from classifiers. The Accuracy metric was calculated as the sum of true positives (TP) and true negatives (TN) divided by the total observations. The "positive" class in the NAC response analysis included patients with pCR, and the "negative" class included patients with RD. False positive (FP) cases were RD cases misclassified as pCR. False negative (FN) cases were pCR case misclassified as RD. Additional confusion matrix performance metrics were used to test for the severity of misclassified cases using metrics such as Accuracy, Precision, Recall (also sensitivity), and F1-score. Accuracy is '(TP+TN) / total cases.' Precision is 'TP / (TP+FP)'. The recall is 'TP / (TP+FN).' Specificity is the number of 'TN / (TN+FP).’

Bibliography

1. Haralick RM, Shanmugam K, Dinstein I. Textural Features for Image Classification. IEEE Transactions on Systems, Man, and Cybernetics. 1973;SMC-3(6):610-21.

2. Jain AK, Farrokhnia F. Unsupervised texture segmentation using Gabor filters. Pattern Recognition. 1991;24(12):1167-86.

3. Wang X, Ding X, Liu C. Gabor filters-based feature extraction for character recognition. Pattern Recognition. 2005;38(3):369-79.

4. Haghighat M. Gabor Feature Extraction GitHub2022 [ February 8, 2022]. Available from: (<https://github.com/mhaghighat/gabor>).

5. Nosaka R, Ohkawa Y, Fukui K. Feature extraction based on co-occurrence of adjacent local binary patterns. Proceedings of the 5th Pacific Rim conference on Advances in Image and Video Technology - Volume Part II; Gwangju, South Korea: Springer-Verlag; 2011. p. 82–91.

6. Ojala T, Pietikäinen M, Mäenpää T. Multiresolution Gray-Scale and Rotation Invariant Texture Classification with Local Binary Patterns. IEEE Trans Pattern Anal Mach Intell. 2002;24(7):971–87.

7. Tamura H, Mori S, Yamawaki T. Textural Features Corresponding to Visual Perception. IEEE Transactions on Systems, Man, and Cybernetics. 1978;8(6):460-73.

8. Julesz B. Textons, the elements of texture perception, and their interactions. Nature. 1981;290(5802):91-7.

9. Kather JN, Weis C-A, Bianconi F, Melchers SM, Schad LR, Gaiser T, et al. Multi-class texture analysis in colorectal cancer histology. Scientific Reports. 2016;6(1):27988.

10. Mathworks. Descriptive Statistics mathworks2021 [Available from: <https://www.mathworks.com/help/stats/descriptive-statistics.html?s_tid=CRUX_lftnav>.

11. Mathworks. Graph and Network Algorithms Mathworks2021 [Available from: <https://www.mathworks.com/help/matlab/graph-and-network-algorithms.html?s_tid=CRUX_lftnav>.

12. Mathworks. Directed and Undirected Graphs Mathworks2021 [Available from: <https://www.mathworks.com/help/matlab/math/directed-and-undirected-graphs.html>.

13. Egeblad M, Nakasone ES, Werb Z. Tumors as Organs: Complex Tissues that Interface with the Entire Organism. Developmental Cell. 2010;18(6):884-901.

14. Ahonen T, Matas J, He C, Pietikäinen M, editors. Rotation Invariant Image Description with Local Binary Pattern Histogram Fourier Features. Image Analysis; 2009 2009//; Berlin, Heidelberg: Springer Berlin Heidelberg.

15. Humeau-Heurtier A. Texture Feature Extraction Methods: A Survey. IEEE Access. 2019;7:8975-9000.
